# Researchers’ and Research Users’ Experiences With and Reasons for Working Together in Spinal Cord Injury Research Partnerships: A Qualitative Study

**DOI:** 10.34172/ijhpm.2021.35

**Published:** 2021-05-11

**Authors:** Femke Hoekstra, Lee Schaefer, Peter Athanasopoulos, SCI Guiding Principles Consensus Panel, Heather L. Gainforth

**Affiliations:** ^1^School of Health and Exercise Sciences, University of British Columbia, Kelowna, BC, Canada.; ^2^International Collaboration on Repair Discoveries (ICORD), University of British Columbia, Vancouver, BC, Canada.; ^3^Department of Kinesiology and Physical Education, McGill University, Montreal, QC, Canada.; ^4^Spinal Cord Injury Ontario, Toronto, ON, Canada.

**Keywords:** Integrated Knowledge Translation, Research Partnerships, Spinal Cord Injury, North America, Capacity Building, System Change

## Abstract

**Background:** Research partnership approaches are becoming popular within spinal cord injury (SCI) health research system, providing opportunities to explore experiences of and learn from SCI research partnership champions. This study aimed to explore and describe SCI researchers’ and research users’ (RU’) experiences with and reasons for conducting and/or disseminating (health) research in partnership in order to gain more insight into potentially ways to build capacity for and foster change to support research partnerships within a health research system.

**Methods:** Underpinned by a pragmatic perspective, ten semi-structured timeline interviews were conducted with researchers and RU who have experiences with SCI research partnerships. Interviews focused on experiences in participants’ lives that have led them to become a person who conducts and/or disseminates research in partnership. Data were analysed using narrative thematic analysis.

**Results:** We identified three threads from participants’ stories: (1) seeing and valuing different perspectives, (2) inspirational role models, and (3) relational and personal aspect of research partnerships. We identified sub-threads related to experiences that participants draw on how they came to be a person who engage in (health) research partnerships, and sub-threads related to participants’ reasons for engaging in research partnerships. While most sub-threads were identified from both researchers’ and RU’ perspectives (eg, partnership successes and failures), some were unique for researchers (morally the right thing to do) or RU (advocating).

**Conclusion:** Using a narrative and pragmatic approach, this study provided a new understanding of SCI researchers’ and RU’ partnership experiences over time. We found that participants’ research partnership experiences and motivations align with components of leadership theories. The findings from this study may be used to inform strategies and policy programs to build capacity for conducting and disseminating (health) research in partnership, within and beyond SCI research.

## Background

**Key Messages**

**Implications for policy makers**

- Policy-makers may use components of leadership theories to build capacity for research partnership approaches within a health research system.
- Promoting an attitude shift on how knowledge should or could be produced may be needed to successfully build capacity.
- Inspirational “role models” may be an effective research partnership capacity building approach.
- Capacity building strategies may focus on encouraging person’s intrinsic motivators rather than extrinsic motivators (eg, rewards).

**Implications for the public**

Conducting and disseminating research in partnership with research users (eg, policy-makers, health practitioners, patients), is a promising approach to improve the use of (health) research in practice. These research partnership approaches are popular within the spinal cord injury (SCI) research system, providing new opportunities to explore experiences of and learn from SCI research partnership champions. We explored and described experiences from SCI researchers’ and research users’ with conducting and/or disseminating (health) research in partnership. We conducted ten timeline interviews with SCI research partnership champions. From participants’ stories, we identified three potential strategies to build capacity for research partnership approaches within an (SCI) health research system. Our findings may be used by policy-makers and other research users to promote and improve research partnership approaches within a health research system.

To address the well-described gap between research and practice or policy,^1,2^ researchers, research users (RU), and funders have increasingly been focusing on ways to enhance the translation of health research to practice or policy settings.^3-6^ Improving knowledge translation (KT) processes is important to ensure that research findings are accessible for people in society (eg, policy-makers, people with disabilities, healthcare professionals). Moreover, improving KT processes may also help ensure research efforts are not wasted (ie, invested time and money).^7^

 A promising approach to enhance KT of health research findings is conducting and disseminating research in partnership with individuals or groups that will use or benefit from the research (ie, RU).^8,9^ Depending on the research, RU may include individuals with lived experiences, clinicians, health and/or social service providers, community organizations, and/or industry partners.^8,10^ Such research partnerships are increasingly being adopted within and outside the health domain as illustrated by the growing body of literature published on different types of research partnership approaches^11,12^ (eg, integrated knowledge translation [IKT],^8,13,14^ community-based participatory research,^15^ patient and public involvement^16,17^) in different areas (eg, public health, social sciences).^9,18^ Because of the many potential benefits that research partnerships can have on the research, community/society and partnership,^9,17,19^ organizations and funding agencies are increasingly promoting this approach.^3,6,20^ However, researchers and RU can perceive many barriers to conducting research in partnership.^14,21-24^ Barriers have been reported on different levels, including the individual-level (eg, lack of understanding about partnership process, lack of partnership skills^14^), the partnership (eg, power imbalances^21^), the research process (eg, required time commitment^22^), and the research system (eg, lack of resources^25^).

 While some guidance for establishing and maintaining effective research partnerships is available in terms of facilitating factors,^13,14,23,25^ tools,^26,27^ guiding principles,^15,28^ mechanisms,^29^ and guidelines,^30^ limited evidence is still available on how, when and why research partnerships can be successfully adopted (or not),^19^ and how to build capacity for research partnerships within a health research system. Furthermore, this raises the questions why and how someone would become a research partnership champion or leader despite the many barriers they may face and limited specific available guidance.

 In response to the lack of specific guidance for health research partnerships,^31^ Bowen et al^32^ explored partnership experiences from senior health personnel. Based on their findings, the authors called for a radical change in how we think about ‘research’ and proposed a ‘multi-system approach’ to address the various barriers and promote effective research partnerships. Following this call, authors from various fields provided additional ideas to promote and support effective research partnerships (eg, principles,^33,34^ strategies,^35^ interventions,^36^ models,^37^ calls to actions^38-40^). While these and other studies^28^ provide some guidance to promoting research partnerships^29^ with specific groups of RU (eg, health personnel, patients, policy-makers), less is known about guidance and capacity building strategies to promote research partnership with different RU (eg, policy-makers, clinicians, patients) in a specific research system. To develop optimal capacity building strategies we need to understand how champions of research partnerships fostered radical change in their own work and in the research system.

 The North American spinal cord injury (SCI) research system is an interesting example of a research system in which a multi-system approach has been used to improve and promote change that can foster meaningful SCI research partnership. In this context, a multidisciplinary team including SCI researchers, RU (eg, people with SCI, representatives from community organizations, clinicians, decision-makers), and funders have been working together to co-develop IKT guiding principles for conducting and disseminating SCI research in partnership using a rigorous and systematic approach.^10^ This initiative was a response to the many poorly implemented SCI research partnerships, in which SCI researchers used a tokenistic approach.^41^ Exploring life experiences or stories from researchers and RU who have been involved in different SCI research partnerships, have advocated for change despite the many barriers to partnership, and value this research approach (ie, “champions”) may provide new insights into ways to build capacity for research partnerships within and outside the SCI research system. Life experiences as well as underlying partnership motivations from champions may be of particular interest as these individuals may have found ways to create radical change and overcome the many barriers they may have faced to adopting a research partnership approach. While (qualitative) research have been carried out focusing on partnership experiences of specific groups (eg, researchers, patients,^42^ healthcare providers, organizations), we are not aware of any research focusing of life experiences or stories from a diverse group of partnership champions (researchers and RU) within a particular research system (ie, SCI) to inform ways to build research partnership capacity across various research areas (eg, health research, social sciences, engineering). Such insights can then be used to develop tailored resources and tools to support and improve (SCI) research partnerships. The aim of this study wasto explore and describe SCI researchers’ and RU’ experiences with and reasons for conducting and/or disseminating research in partnership in order to gain more insight into potential ways to build capacity for and foster change to support (SCI) research partnerships within a health research system. The underlying research questions were:

- How and why do researchers and RU become champions/leaders of SCI research partnerships, despite the many barriers they may face?
- What are researchers’ and RU’ experiences/approaches of becoming a SCI research partnership champion?

## Methods

###  Study Overview and Research Perspective 

 This qualitative study was based on interviews with SCI researchers and RU. This paper includes a short overview of the methods. Additional information on our study procedures including the COREQ reporting checklist^43^ are available in Supplementary files 1 and 2 and on Open Science Framework (OSF, https://osf.io/n5r4h/). We approached this study from a pragmatic perspective.^44^ When adopting a pragmatic approach, the primary aim of the research is to use research to solve practical “real-world” problems. Pragmatism follows an ontological relativist paradigm (ie, truth is uncertain, is based on context and based on what works in certain contexts) and emphasizes the practical outcomes of the knowledge within a particular situation (ie, outcomes are not necessarily generalizable) rather than seeking a single truth. In line with a pragmatic perspective,^45^ we adopted an IKT approach to enhance the relevance and usefulness of our findings.^46^ A multidisciplinary panel including SCI researchers, people with SCI, and other SCI RU (eg, clinicians, organizations, funders) was established to co-create the first IKT guiding principles for the SCI research system. The panel defined IKT as “*the meaningful engagement of the right RU at the right time throughout the SCI research process*.” The panel recommended that the IKT guiding principles and dissemination efforts should be informed by interviews with partnership champions (researchers and RU). Panel members were engaged in design of the study, recruitment procedures, data interpretation, and dissemination of the findings. Supplementary files 3 and 4 include details on panel members’ names, organizations and roles and the collaborative research activities.

###  Participants and Sampling 

 Participants were recruited via purposive sampling using maximum variation and criterion-based sampling strategies.^47^ To include a variety of partnership experiences we looked for variation in the following characteristics: gender, age, research area, and roles in the partnership (researcher and RU). While maximum variation sampling contributed to including a variety of SCI research partnership experiences, criterion-based sampling contributed to including participants who share common inclusion criteria based on certain experiences and characteristics. The inclusion criteria for participants were: (1) being 18 years and older, (2) having experiences with conducting and/or disseminating research within an SCI research partnership, and (3) having positive attitude towards engaging SCI community members in the research process.

 Panel members’ personal networks were used to identify potential participants. Interested participants completed a short online survey including general demographic information.

###  Data Collection 

 After participants completed the survey, the first author (FH) contacted the participants to schedule one semi-structured interview. Prior to the interview, participants were asked to create a timeline of moments or experiences in their life that have led them to become a person who conducts and/or disseminates research in partnership. From this information, the first author (FH) created a personalized timeline.

 The interview session included two parts. Part 1 focused on describing and understanding participants’ experiences with SCI research partnerships, which is the focus of the current paper. Part 2 focused on principles and strategies of SCI research partnerships, which was described in a separate paper. The first part of the interview included questions about participants’ general experiences with (SCI) research and partnerships. Participants’ personalized timeline, focusing on their temporal experiences, was used as an artefact to guide the interview session.^48^ Two separate interview guides were constructed, each tailored towards participants’ primary role (researcher or RU). The interviewer invited the participants to elaborate on how they came to be a person who conducts and/or disseminates research in SCI research partnerships. The interview guides are available on OSF (https://osf.io/n5r4h/). Interviews were conducted using an online videoconference interface and lasted on average 74 minutes (range: 39-107 minutes).

###  Data Analysis 

 Interviews were audio recorded and transcribed verbatim. Names and organizations were anonymized and participants were given pseudonyms. A narrative thematic analysis was conducted using steps described by.^49,50^ Narrative thematic analysis differs from other analyses because it focuses on themes or threads within a story instead of across stories. After transcribing and organizing the data, the first author (FH) started the analysis with a period of narrative indwelling, which included listening to the audio-recordings, reading the transcripts and timelines, and making notes. Next, FH identified narrative threads (ie, “patterns that run through each story”) within each transcript by identifying fragments of the story, highlighting key phrases in the transcript, and summarizing data in a search for underlying meaning. For each participant, a short narrative account was written that illustrated participants’ key experiences to help identify threads. Next, FH identified threads related to participants’ SCI research partnerships experiences by reviewing the complete data set (transcripts, timelines, notes, and summaries). In addition, we identified narrative sub-threads focusing on participants’ SCI research partnership experiences and their reasons to engage in research partnerships. The narrative threads were then linked to existing literature to enhance the usefulness of our findings. The threads and sub-threads were finalized after several discussions with co-authors (LS, PA, HLG), who acted as “critical friends” throughout the analyses process.^47^ Further information on these reflective discussions is described in Supplementary file 5. Analyses were supported by NVivo 12.

###  Methodological Rigour

 Different strategies were used to enhance the quality and methodological rigour of our study.^51^ First, we used a narrative approach, in which the interviews were guided by participants’ timelines and personal stories. The timelines allowed us to gather data connected to how participants made meaning of becoming involved in research partnership projects, which contributed to a rich understanding. We also linked the threads to existing literature, which further enriched our findings. This study was also conducted in collaboration with RU (ie, SCI IKT Guiding Principles Panel) using an IKT approach which contributed to the worthiness of the topic. The worthiness of the topic is further illustrated by the fact that the research question underlying this study (ie, development of IKT Guiding Principles for the SCI research system) came from the SCI community members itself. Third, LS, PA, and HLG acted as a “critical friends” throughout data analyses, allowing for alternative interpretations and further reflection, which contributed to meaningful coherence of the study findings. Panel members had the opportunity to provide feedback on the findings contributing to further enhanced coherence and relevance of the study.

## Results

 Ten participants (gender: 7 women and 3 men) with ages ranging between 34 and 57 years (mean ± SD: 46 ± 7) were included. Five participants were primarily working as academic researchers in different fields (eg, social sciences, health sciences, health promotion, engineering) at North American universities. Four participants (researchers or RU) had a clinical background (eg, physiotherapist, nurse, occupational therapist). Five participants were affiliated with an SCI community or advocacy organization and three participants had lived experiences with SCI. Seven participants provided views from different perspectives (eg, researcher *and* clinician, representative of community organization *and* person with SCI, researcher *and* person with SCI). To protect participants’ identities further demographic details are not provided.

 From participants’ stories, we identified the following three narrative threads:

1. Seeing and valuing different perspectives
2. Inspirational role models for RU engagement in SCI research
3. Relational and personal aspects of research partnerships and its evolvement over time

Within each thread, we identified sub-threads related to experiences that participants drew on how they came to be a person who conducts and/or disseminates research in SCI research partnerships, and sub-threads related to participants’ reasons engage in SCI research partnerships. Figure summarizes the identified threads and sub-threads. Threads are described below and illustrated with quotations from participants.

**Figure:**
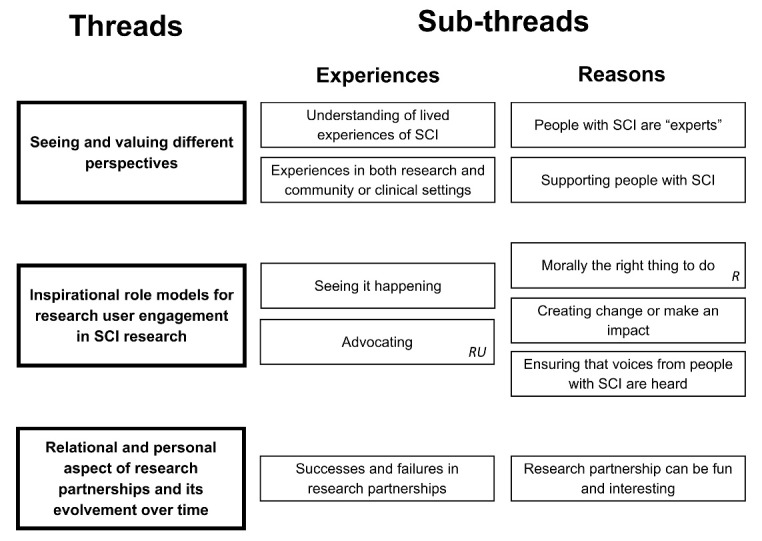
An Overview of the Three Narrative Threads and Related Sub-threads Identified From Participants’ Stories on SCI Research Partnership Experiences.R or RU indicates that the sub-thread was unique for researchers (R) or RU. Abbreviations: SCI, spinal cord injury; RU, research users.

###  Seeing and Valuing Different Perspectives 

 The first thread relates to seeing and valuing different perspectives. Within this thread we identified two sub-threads related to participants’ experiences (experiences in both research and community or clinical settings, understanding of living with SCI) and two sub-threads to participants’ reasons (people with SCI are “experts,” supporting people with SCI).

####  Understanding of Lived Experiences of Spinal Cord Injury 

*“It became really important to me to understand it [research] better through people’s lived experience.” *

 Olivia and other participants (researchers and RU) drew on their experiences with working alongside people with SCI or living with an SCI. Researchers mentioned that by working closely with people with SCI as a clinician and/or volunteer, they have learned more about what it is like to live with an SCI, and in turn better understand the challenges people with SCI face in life. It also helped them see and value different perspectives, which transferred into their SCI research projects. While researchers acknowledged the importance of interacting with people with SCI, for Tyler and Audrey, it was initially very uncomfortable. Audrey told a story about her first experience working alongside people with SCI. She had the opportunity to do an internship at the local university in which she assisted with data collection of a SCI research. This internship was an overwhelming experience for her and concluded that she did not want to do SCI research:

*“At that time it was completely overwhelming, because I had never seen a person in a wheelchair and now I was helping a person with a wheelchair get into an MRI machine. I remember at the end of the internship, the PhD-student said “what is the biggest lesson you are going to take from this experience?” And do you want to know what I said? “I am uncomfortable with people with SCI.” That is what I told him, and I never wanted to do research, it is too hard, and is kind of boring.” *

 However, later in her training, she volunteered alongside people with disabilities and decided to do a PhD in SCI research:

*“I think I just needed to feel out of my comfort zone. I think that experience stayed with me and as I gained more confidence in research and more confidence in the population by working with them, I felt better about that. It’s just ironic that what I ended up doing what I said I didn’t want to do, is actually what I did.” *

 Samantha, a RU with SCI and academic background, emphasized throughout the interview her unique skills of being able to see things from different perspectives.

*“Because I’ve got that research background I can wear multiple hats. I can sit at a meeting and see things from the perspective of a researcher as well as the perspective of someone living with an SCI. Plus I can see it from the perspective of the participant in the study and I can see things from the perspective of the funder.” *

 Samantha recognized that with her background, she has a unique skill set that she can bring to the table, which motivates her to engage in SCI research partnership projects that are meaningful to her.

####  Experiences in Both Research and Community or Clinical Settings

*“It would be a fun way for us [researchers and people with disabilities] to spend time together and I went to volunteer and it was like a huge transformational experience because prior to that I did not have much experience with working with people with disabilities.” *

 As described in the previous section, Audrey emphasized how her volunteer work has shaped her as a researcher who wants to learn from people with disabilities and who wants to see the practical application of her research findings in the community. Audrey and other participants(researchers and RU) talked about their career-related experiences, within and outside academia, that have helped them to see and value things from different perspectives. To illustrate, participants in our study included researchers with clinical backgrounds (eg, physiotherapist, nurse) and SCI RU with academic backgrounds (bachelor, master or PhD). Participants mentioned that being able to “*wear multiple hats*” facilitated the collaborative research activities. For example, Charlotte started a job in research after she has worked as a clinician with people with SCI for many years. When she started her research job, she was very naïve about the research process:

*“I would say I was fairly naïve about research particularly, how it gets disseminated was the most surprising thing to me when I moved into more of a research role. […] I guess that was somewhat of an “ah-ha” moment like this [research findings] is going to just sit in a journal somewhere? So that was frustrating to me.” *

 Through her knowledge and understanding of living with an SCI, as well as her clinical experience, she was able to feel like she improved the dissemination of research findings.

####  People With Spinal Cord Injury Are “Experts” 

*“I remember being at this meeting and learning so much from the people on the team that had an SCI, so the majority of the presenters were either healthcare professionals who work with people with a disability, or they were individuals with a disability and it just so happened that they were people with SCI.” *

 In this quote, Audrey drew upon her first research partnership project. Audrey and other participants (researchers and RU) valued the knowledge and lived experiences of people with SCI. They see people with SCI and those who work alongside people with SCI (eg, clinicians) as “experts,” who can provide valuable input to the research process. Participants perceived that by engaging these “*experts*” in the research process, it improved the quality of the research.

 Olivia, who became introduced into SCI research later in her research career, realized that she never really had an understanding of what it meant for people to live with an SCI or how they conceptualized the impact of research outcomes.

*“It became really important to me to understand it better through people’s lived experience. […] If you don’t have the person’s perspective, how do you know what it is you’re developing is going to be something that they actually want?*”

 In her transition to an SCI researcher, it became important for her to better understand SCI through people’s lived experiences. She felt she was able to do better research and create better interventions by engaging people with SCI throughout the entire research process.

####  Supporting People With Spinal Cord Injury

*“So usually when you do research you don’t have very much of a social benefit to society. [By hiring people with SCI], we were actually able to give back to the community.” *

 Olivia, as a senior researcher, felt that she was able to give something back to the SCI community by hiring people with SCI as part of the research team because of their unique expertise. Similarly, the majority of the participants (researchers and RU) mentioned that an important reason to engage in SCI research partnerships is that they wanted to support or help people with SCI. Participants explained it as important, because “helping people with SCI” was often the common goal/vision of the research partnership. This “caring” aspect was especially highlighted by participants with clinical backgrounds. For example, researcher Audrey mentioned that she always had a passion to help people with SCI and improve their quality of life. Conducting research in partnership with community members was the type of research in which she could incorporate her passions.

###  Inspirational Role Models for RU Engagement in SCI Research 

 The second thread relates to inspirational role models for RU engagement in SCI research and beyond. Within this thread we identified two sub-threads related to participants’ experiences (seeing it happening, advocating) and three sub-threads related to participants’ reasons (morally the right thing to do, creating change, ensuring that voices from people with SCI are heard).

####  Seeing It Happening

*“That’s where I got to see the importance of how much insight community members have and how much they can drive research questions and the value of really integrating them into the research process. It’s still in my memory, it’s still very vivid to me that experience and seeing how it works and how to see it live.”*

Tyler and other researchers and RU drew upon stories in which they felt inspired by other leaders in SCI research partnerships through attending SCI collaborative conferences, partnership meetings, and/or lectures about research partnership approaches. Participants drew “*attending a joint SCI collaborative conference*” as key experiences on their timelines. They mentioned that they felt inspired by seeing how SCI researchers, people with SCI, and other RU were jointly attending similar sessions and discussions.

Researchers, in particular, talked about the value of their supervisors or peer-researchers and how they acted as role models. Their stories showed how attending collaborative conferences or meetings changed how participants thought about the research process. To illustrate, Tyler attended a research partnership meeting for the first time during his postdoctoral research. In this meeting, led by his supervisor, SCI researchers and RU were collaboratively discussing the findings of a research project. By attending that meeting, he was able to see how a research partnership could work, and became excited about doing this type of research. In that same year, Tyler attended a collaborative conference, which inspired him because it gave him the opportunity to meet and talk to people with SCI and get a better understanding of what it is like to live with an SCI. He remembered that attending this conference “*was not so much about the conference, but more about getting introduced to people with SCI and understanding more about what it’s like to experience a SCI*.”

 Audrey highlighted the value of the mentorship and training she received throughout her academic traineeship. She was able to see how her supervisors, who acted as role models, involved people from the SCI community in their research. As such, doing research in partnership was for her the “default:”

*“It was never really, like doing a research project without asking a person with SCI what they thought was almost like: “Does that exist? People do that? People don’t engage the community? Like how do you know what they need or what they want, if they don’t have a say, or if you don’t ask them?” Because I came from a practical stand point, there was no other way [for me] of doing research.”*

 RU also drew strongly on attending collaborative conferences. Valerie and Samantha, who both have an SCI, mentioned that attending these conferences was both inspiring and empowering. Valerie explained that attending one of these conferences has changed the way she thought about equitable and meaningful RU engagement. She mentioned that the conference was fruitful for her because it changed her mindset about how to engage people, participants and communities, in research projects. She learned about how people with disabilities could be engaged in research projects in a scientific way that also ensures that their voices are being heard. Besides being inspired by other role models, Valerie and Samantha’s stories illustrated how they acted as role models for other researchers and RU. For example, Samantha stated:

*“I’ve been involved for so many years, as a participant, as a research assistant, then doing my Masters degree. The research community knows me, they see me. I’ve been visible and over the years it’s almost like experience shows, you know. I mean when you sit on meetings and you actually contribute in a meaningful way they [researchers] can see that I was the RU that contributed, it’s a snowball effect. They know you the next time, because you might have something very interesting to say, to contribute, to the project that they may not have thought of.” *

####  Advocating 

*“I was involved with creating peer advocacy materials and workshops and encouraging kids with spinal cord paralysis to learn how to take on advocating for themselves and their health conditions.”*

 Valerie and other RU talked about their own advocating experiences for RU engagement in SCI research. Participants fulfilled advocacy roles as part of their jobs, both within and beyond research. For example, Valerie worked as an advocate to encourage kids/youths with different kinds of conditions to be (more) involved in their own healthcare. From her own experiences with receiving medical care as a person with SCI, Valerie has noticed the value of being engaged as a patient in your own care.

*“That was a really formative experience for me, because I think I realized the value of being a really engaged patient versus someone who wasn’t, the value for the person, the value for their care, the value for those who love them, and the people who treat them.”*

 While Valerie started her career as an advocate for patient engagement in healthcare, she is currently fulfilling an advocating role for RU engagement in SCI research. With her academic background and her interests in participating and conducting (health) research, the shift from patient/RU engagement in healthcare to research, was a natural transition.

####  Morally the Right Thing to Do

*“Maybe it’s around axiology. I guess to me there is a moral dimension to why we do this. You really believe that we shouldn’t do research on people, we should do research with people.”*

Thomas and other researchers believe that research should be done “*with* participants not just *on* them.” Adhering to a pragmatic process, Thomas sees this morally driven process as a way to support research that is more effective for participants and communities.

*“Every choice is a compromise, yes it adds complexity, but by the same token I think it is more moral and I do think you get better outcomes. I think you do a better job, if you are involving people throughout the process.”*

 Like other participants, Thomas denotes the complexities and compromises that come from including RU in the research process. While the feeling of being the expert and prescribing research agendas on communities is lost, the feeling of purpose and making a difference seems to be worth the while.

*“And then hopefully you’re able to mobilize the findings better and cause change. So for me, I think it is worth that cost, that complexity and time right. So its complexity and time. Because you have to be willing to do both.”*

 Thomas highlighted the importance of involving RU throughout the research process. As he described this is not always easy and often takes time, but for him worth it in regards to feeling like you are actually able to see research and results make change. Embracing the research partnership approach offers a better way to do research. It allows critical friends, in this case community members, to be involved in the entire research process. It also enables a closer, in depth interpretation of how individuals make meaning of having living with an SCI.

####  Create Change or Make Impact 

*“I was doing data entry from paper questionnaires. That’s where I start seeing that you can do research that can potentially have a direct impact on certain individuals.”*

Tyler and other participants (researcher and RU) highlighted that they engage in SCI research partnerships because they want to do relevant and useful research, which can “*make a difference*” or “*create change*” on an individual, organization, and/or system. Especially participants with a clinical/health background (eg, physiotherapist, nurse, occupational therapist, exercise therapist) talked about the importance of implementing the research findings in community or clinical settings. For example, Naomi worked as a clinician and became interested in research because she wanted to better integrate evidence into her clinical practice. It shows her ability to transact with the research within a local context.

*“I think through being a clinician first [..]. My goal of going into research was to make sure I could better integrate evidence into clinical practice.”*

 To be able to make a difference, participants mentioned that they wanted to ensure that the research they are involved in is relevant and useful. Participants provided examples from basic science and practical-focused research projects. While the majority of participants mentioned that they wanted to “create a change” or make an impact, Arthur emphasised his “curiosity, interest driven” as an important reason. Pragmatically, Arthur’s research interest is driven by his own lived experiences in SCI.

####  Ensuring that Voices From People With SCI Are Heard 

*“…bringing the power and the voice of the community to help us [people with SCI] define and address what the needs are, what the areas of focus for research should be, and advocacy. The power of taking that research and putting it into practice, fighting for policy changes. So it’s really important that we bring everyone together. Everyone has a particular skill set that they can bring to the table.”*

 Samantha, a person with SCI, emphasized throughout her stories the importance to ensure that voices from people with SCI are heard throughout the research process. In this context, she also referred to the idea of “nothing about us, without us.” For Samantha and other participants (researchers and RU) this was one of the underlying reasons to engage in and/or advocate for SCI research partnerships.

 While participants highlighted the importance to incorporate voices from people with SCI in research, they also talked about associated challenges. For example, Samantha explained that people with SCI could feel intimidated when they are invited to join a research project:

*“They feel very intimidated and they feel that their voice doesn’t count or is not heard or not respected or not valued, but if we can give them some basic knowledge [on the research process], so they have a basic understanding, I think they would feel more empowered to be stronger participants in the decision-making process.”*

 Samantha continued her story on her involvement in the development of tools aiming to educate and empower people in the SCI community to engage in research projects. She explained that these tools could address some of the challenges related to research partnership approaches.

###  Relational and Personal Aspects of SCI Research Partnerships 

 The third thread relates to relational and personal aspects of SCI research partnerships and its evolvement over time. Within this thread we identified one sub-thread related to participants’ experiences (successes and failures in research partnerships) and one sub-thread related to participants’ reasons (research partnerships can be fun).

####  Successes and Failures in Research Partnerships 

*“I think there is as much learning for researchers how to work with community members, as for community members how to work with researchers.” *

 Although participants (researchers and RU) mainly talked about their successful partnership experiences, they also provided examples of ‘tokenism’, ‘less successful’ or ‘poor’ partnerships. For example, Tyler elaborated on challenges he experienced in his first SCI research partnership. To develop a relationship, he reached out to a community organization, who was very excited about the idea to collaborate. Although his community partners were engaged throughout the research process, he perceived that “*They [community partner] became a little less collaborative for a few months [after the results were presented to them]*.”

 Throughout Tyler’s story, he provided examples of how he was able to learn from his previous partnership experiences. For example, in next projects he ensured that roles and responsibilities of all partners were clear from the start of the project. By reflecting on his collaborative research activities and learning from his previous research partnership challenges, he was able to improve his partnership skills. While researchers experienced several challenges (eg, it takes time, logistics and planning of meetings are complicated, dealing with different opinions) when conducting research in partnership, they also indicated that these “challenges are worth overcoming.”

 When sharing participants’ successes and failures, participants also indicated they were interested in figuring out what the best way is to engage RU in the research process. From researchers’ perspectives, participants seem to be very aware of how they work in partnership, how they build and maintain their relationship, and how they can do better. As illustrated, researcher Tyler was very reflective and wanted to learn from previous projects. RU talked about ways to encourage (other) people with SCI to participate in the research process. For example, Valerie, a RU with an SCI, stated:

*“Why would people come to the next meeting? What would make it worthwhile to give them the maximal opportunity to contribute? What is expedient for researchers is not as engaging or expedient for the general public. I still don’t have like brilliant solutions for it. [...] I think we’re still trying to learn.” *

####  Research Partnerships Can Be Fun and Interesting 

*“This is cool! And this is fun! And this is the type of research I want to do, and this is really fun! The bouncing of ideas and the interaction you have with other researchers and with other community members, the breadth of knowledge that gets derived from so many minds, brainstorming it, thinking about the next steps in the future I thought that was really… I guess excitement is a good emotion for that moment.” *

Tyler and other participants (researchers and RU) drew upon the fun and interesting aspects of engaging in SCI research partnerships. Researchers also mentioned that working in research partnerships is rewarding. While researchers highlighted the enjoyment of being part of a research partnership, RU indicated that they engage in research because they were interested in the research topic. It illustrates participants’ sense of autonomy related to their decisions to engage in research partnerships. Thomas talked about the relational and personal aspect of doing research in partnership and linked it to researchers’ personality types (see Box 1).

**Box 1. Summarizing Quote From a Research Partnership Champion (Thomas)**

“Part of it is being open to collaboration. I do think it’s a different way of thinking, and you do have to relinquish control. For a lot of researchers that’s not the personality type they come from. I think a lot of researchers are more type A personalities. They like to bend the environment around them to their own wills. You can accomplish great things that way, but you can also become Voldemort. Being a participatory action researcher, I don’t know if it’s more type A, or type B. You have to be better able to handle uncertainty, because you don’t really know what’s going to happen. You need to find people you want to work with. [..] Its relationships that is what this type of research is all about. It’s all relational, in a system’s perspective and on individual level. And that’s a big challenge. Some people are not designed for that kind of social interaction, and many of us are researchers. A lot of people went into research so they wouldn’t have to do that. But I think it’s really interesting when you generate that kind of team. Because then it becomes, yes you don’t know what’s going to happen, but you are just happy to be part of the process. Because you trust these people, you like working with them. [...] These people are experts that you’re working with and they’re experts in ways you will never be an expert. Because they have those experiences in that condition.”

## Discussion

 This is the first study that explored life experiences from SCI research partnership champions in order to gain more insight into effective ways to build capacity for and foster change to support research partnerships. We identified three narrative threads related to (1) seeing and valuing different perspectives, (2) inspirational role models, and (3) personal and relational aspects of research partnerships. While most sub-threads related to participants’ experiences and reasons were both identified from a researcher and RU perspective, some were unique for researchers (“people with SCI are experts”) or RU (“advocating”). By linking our findings to existing literature and (leadership) theories (eg, transformational leadership theory,^52^ self-determination theory^53^), we identified three potential approaches to build capacity for and foster radical change to support research partnerships within a (health) research system.

###  Attitude Shift on How Knowledge Should or Could Be Produced 

 The first approach relates to the attitude shift of valuing different perspectives and knowledge. Participants indicated that they see people with SCI as “experts,” who could add valuable knowledge and expertise to research projects. This finding indicates that engaging in successful research partnerships require a view around research in which knowledge is not simply produced by people with academic backgrounds (bachelor, master or PhD), but through interactions alongside people with lived experiences (eg, SCI or other disability or chronic disease) and/or other RU.^54^ As such, aligning with previous literature,^32,55,56^ building capacity for research partnerships within a research system may focus on promoting such *an attitude shift*. For SCI researchers, this may indicate that people with lived experiences in SCI are recognized as “experts” who are valued as co-researchers,^42^ not simply as participants. Introducing this constructivist notion, can bump with some scientific paradigms, and may therefore be introduced when people are early in their career. We see this as empowering individuals with SCI and those who have worked alongside people with SCI (eg, clinicians),^57^ which promotes meaningful contributions to SCI research projects from those who have long been excluded. Pragmatically, offering workshops and/or training to researchers, trainees and RU about how and why research could be conducted and disseminated in partnership may be a way to promote such an attitude shift on how knowledge could be produced.^32^

###  Inspirational Role Models 

 The second approach relates to inspirational role models for research partnerships. Participants provided several examples of how they were inspired by their supervisors or peers (“role models”) to meaningfully engage in research partnerships. They also provided illustrative examples of how these experiences inspired them to follow in their peers’ footsteps and become role models who inspire others. These findings resonate with transformational leadership theory,^52^ particularly with the *inspirational motivation* (ie, inspire and motivate others with enthusiasm and positivity) and *idealized influence *(ie, leaders are admired and act as role models) components of the theory. Several participants’ stories, characterized them as “transformational leaders” with *idealized influence* that was driven by moral reasons to create change. The importance of inspirational “role models” and “leaders” in creating system-level changes has been described in previous literature^58,59^ and aligns with tenets of Roger’s diffusion of innovation^60^ theory and social cognitive theory.^61^ Based on our findings, we suggest that inspirational “role models” are key asset to adopting a research partnership approach, and could be a promising approach to build capacity among researchers and RU. The sharing of these powerful stories from inspirational role models may provide a novel approach to effective KT.^62,63^

###  Relational Research Partnership Skills 

 The third approach relates to the relational aspects of research partnerships. Participants’ stories revealed that those who engaged in research partnerships were highly motivated and willing to improve their relational research partnership skills (eg, building relationships). Participants were reflective and thoughtful on how they work alongside partners, which resonates with the *intellectual stimulation *(ie,stimulate to be creative and innovate) component of transformation leadership theory.^52^ Furthermore, they talked about the enjoyment of working in partnership suggesting that participants may be *intrinsically motivated* to engage in research partnerships. According to Self-Determination Theory,^53^ three psychological needs (autonomy, competence, relatedness) influence whether a person is intrinsically motivated to engage in a certain behaviour. This may suggest why researchers and RU who support these three needs may be more successful in research partnerships. To illustrate, participants were able to make their own decisions to engage in research partnerships (autonomy), participants felt socially connected to the SCI community (relatedness), and participants were trained in research partnership skills (competence). As such, building capacity for relational research partnerships may focus on encouraging intrinsic motivators to engage in research partnerships rather than extrinsic motivators (eg, reward systems or mandated partnerships). Relational approaches to research can bump with dominant research paradigms.^64^ It is therefore important to provide not only additional training, but also additional advocacy for an approach that values relationships as an important aspect of the research process, aligning with Bowen and colleagues’ call to reimagine research.^32^ Organizing collaborative meetings in which researchers and RU have opportunities to interact with each other may be a promising advocacy strategy.

###  Scientific and Practical Implications 

 This study has important scientific and practical implications. For the KT and research partnership literature, the findings provide new insights into ways to understand successful research partnership behaviours and explore potentially effective research partnerships capacity building approaches that can foster radical change in a research system. Our findings may suggest that effective research partnership behaviours, as described by SCI research partnership champions, align with components of transformational leadership theory (inspirational motivation, idealized influence, individualized consideration, intellectual stimulation). To further advance the science of KT and research partnerships, future research should focus on understanding effective research partnership behaviours by using leadership theories,^52^ implementation,^65^ KT,^66^ and/or RU engagement frameworks.^67^

 We found that participants’ reasons to engage in research partnerships were mainly related to moral and relational dimensions, which aligns with previous literature.^30,68,69^ This finding may suggest that groups and/or organizations that want to promote and support research partnership approaches may want to use a transformational leadership approach^52^. Conversely, this finding may suggest that a transactional leadership approach, which focuses on offering (financial) rewards or mandating practice, may not be an ideal way for research partnership capacity building. This finding may have important implications for funding agencies and other organizations (eg, universities, community organizations) that want to promote research partnership approaches and/or develop supporting resources. Our findings caution against mandating or incentivizing research partnership approaches. While our findings align with previous partnership guidance and call to actions,^30,70^ future research is needed to explore effective ways to build capacity for research partnership within and beyond the SCI research system, from a transformational perspective.

###  Limitations

 Some limitations must be acknowledged. First, our sample did not include people who do not have experiences with research partnerships. While the focus on partnership champions’ experiences aligned with our research questions, we do provide suggestions in our discussion section for ways to build capacity for and foster radical change to support research partnerships. We do not know if these approaches are effective and if they resonate with people who do not have any experiences with research partnerships and who have not (yet) created radical change. As such, our suggestions should be applied with caution and more research on this topic is needed. Second, our sample included only RU with university backgrounds. Although our sample did not represent people with SCI without a university background, participants talked about the importance to offer education and training to people with SCI who want to engage in research partnerships. Third, the interviews did not focus on co-creation of participants’ timelines, which is considered as a benefit of timeline interviews.^48^ Instead, the interviewer created personalized timelines prior to each interview based on participants’ responses, which were then used to guide the session. This approach allowed us to limit our interviews to one session, as a multi-session interview would be more time-consuming for participants. Despite these limitations, we were able to collect a rich data set from a diverse group of research partnership champions, which provided valuable practical insights on a variety of partnership experiences. Lastly, we were unable to describe and explore certain narrative types, because such an approach would breach participants’ confidentiality.

## Conclusion

 Using a narrative and pragmatic approach, this study provided a new understanding of the experiences of SCI researchers’ and RU’ partnership experiences over time. The findings revealed that participants’ research partnership experiences align with leadership theories. We identified three potential ways to build capacity for and foster radical change to support research partnerships within a (health) research system: (1) promoting attitude shift on the value of co-production of knowledge, (2) using inspirational “role models,” and (3) encouraging intrinsic motivators for research partnership engagement. The findings from this study may be used to inform strategies and programs to build capacity for conducting and disseminating research in partnership, within and beyond SCI research.

## Acknowledgements

 SCI Guiding Principles Consensus Panel includes Kim Anderson (NASCIC), Hugh Anton (ICORD, UBC Medicine), John Chernesky (Praxis Research Institute, NASCIC), Susan Forwell (ICORD, UBC Occupational Therapy), Jocelyn Maffin (SCI BC), Kathleen Martin Ginis (UBC, ICORD), Christopher B McBride (SCI Canada, SCI BC), W. Ben Mortenson (UBC, ICORD), Rhonda Willms (ICORD, GF Strong, UBC Medicine). The authors would like to thank Michael Kennefick, Clayton March, Kerri Huhn, Aswathy Kumanan, and Alexandra Jab for assisting in transcribing the data.

## Ethical issues

 This study has been approved by the Behavioural Research Ethics Board of the University of British Columbia Okanagan (H18-01680).

## Competing interests

 Dr. Gainforth reports grants from Michael Smith Foundation for Health Research Scholar Award (Dr. Gainforth: 16910), International Collaboration on Repair Discoveries (Dr. Gainforth), and Social Sciences and Humanities Research Council of Canada (890-2018-0044) during the conduct of the study. Authors (HLG, PA) and the SCI Guiding Principles Consensus Panel play a leadership role within the SCI Research System. Additionally, the panel and all authors are funded to conduct research using and/or investigating an integrated knowledge translation approach.

## Authors’ contributions

 All authors, including the panel, contributed to the conception and design of the project. FH was involved in the acquisition of the data. All authors, including the panel, contributed to analysis and interpretation of the data. FH drafted the first version of the manuscript. All authors and the panel provided edits and critical revisions of the manuscript. HLG and the panel contributed to obtaining funding. HLG supervised FH throughout the study. All authors and the panel approved the manuscript.

## Funding

 This study is supported in part by the Social Sciences and Humanities Research Council of Canada (890-2018-0044), a Michael Smith Foundation for Health Research Scholar Award (Dr. Gainforth: 16910), and by the International Collaboration on Repair Discoveries.

## Supplementary files

Supplementary file 1. Research Team and Study Procedures.

Supplementary file 2. COREQ Reporting Checklist.

Supplementary file 3. Members of the SCI Guiding Principles Panel.

Supplementary file 4. Research User Engagement in the Qualitative Research Study.

Supplementary file 5. Role of Critical Friends in the Analyses Process.

## References

[1] Glasgow RE, Lichtenstein E, Marcus AC (2003). Why don’t we see more translation of health promotion research to practice? Rethinking the efficacy-to-effectiveness transition. Am J Public Health.

[2] Morris ZS, Wooding S, Grant J (2011). The answer is 17 years, what is the question: understanding time lags in translational research. J R Soc Med.

[3] Tetroe JM, Graham ID, Foy R (2008). Health research funding agencies’ support and promotion of knowledge translation: an international study. Milbank Q.

[4] Oborn E, Barrett M, Racko G (2013). Knowledge translation in healthcare: incorporating theories of learning and knowledge from the management literature. J Health Organ Manag.

[5] Robinson T, Bailey C, Morris H (2020). Bridging the research-practice gap in healthcare: a rapid review of research translation centres in England and Australia. Health Res Policy Syst.

[6] McLean RKD, Graham ID, Tetroe JM, Volmink JA (2018). Translating research into action: an international study of the role of research funders. Health Res Policy Syst.

[7] Macleod MR, Michie S, Roberts I (2014). Biomedical research: increasing value, reducing waste. Lancet.

[8] Canadian Institutes of Health Research (CIHR). Guide to Knowledge Translation Planning at CIHR: Integrated and End-of-Grant Approaches. Ottawa: CIHR; 2012.

[9] Hoekstra F, Mrklas KJ, Khan M (2020). A review of reviews on principles, strategies, outcomes and impacts of research partnerships approaches: a first step in synthesising the research partnership literature. Health Res Policy Syst.

[10] Gainforth HL, Hoekstra F, McKay R (2021). Integrated knowledge translation guiding principles for conducting and disseminating spinal cord injury research in partnership. Arch Phys Med Rehabil.

[11] Nguyen T, Graham ID, Mrklas KJ (2020). How does integrated knowledge translation (IKT) compare to other collaborative research approaches to generating and translating knowledge? Learning from experts in the field. Health Res Policy Syst.

[12] Jull J, Giles A, Graham ID (2017). Community-based participatory research and integrated knowledge translation: advancing the co-creation of knowledge. Implement Sci.

[13] Lawrence LM, Bishop A, Curran J (2019). Integrated knowledge translation with public health policy makers: a scoping review. Healthc Policy.

[14] Gagliardi AR, Berta W, Kothari A, Boyko J, Urquhart R (2016). Integrated knowledge translation (IKT) in health care: a scoping review. Implement Sci.

[15] Israel BA, Parker EA, Rowe Z (2005). Community-based participatory research: lessons learned from the Centers for Children’s Environmental Health and Disease Prevention Research. Environ Health Perspect.

[16] Boote J, Wong R, Booth A (2015). ‘Talking the talk or walking the walk?’ a bibliometric review of the literature on public involvement in health research published between 1995 and 2009. Health Expect.

[17] Brett J, Staniszewska S, Mockford C (2014). Mapping the impact of patient and public involvement on health and social care research: a systematic review. Health Expect.

[18] Slattery P, Saeri AK, Bragge P (2020). Research co-design in health: a rapid overview of reviews. Health Res Policy Syst.

[19] Gagliardi AR, Kothari A, Graham ID (2017). Research agenda for integrated knowledge translation (IKT) in healthcare: what we know and do not yet know. J Epidemiol Community Health.

[20] Gainforth HL, Baxter K, Baron J, Michalovic E, Caron JG, Sweet SN (2019). RE-AIMing conferences: evaluating the adoption, implementation and maintenance of the Rick Hansen Institute’s Praxis 2016. Health Res Policy Syst.

[21] Brush BL, Mentz G, Jensen M (2020). Success in long-standing community-based participatory research (CBPR) partnerships: a scoping literature review. Health Educ Behav.

[22] Bird M, Ouellette C, Whitmore C (2020). Preparing for patient partnership: a scoping review of patient partner engagement and evaluation in research. Health Expect.

[23] Drahota A, Meza RD, Brikho B (2016). Community-academic partnerships: a systematic review of the state of the literature and recommendations for future research. Milbank Q.

[24] Oliver K, Kothari A, Mays N (2019). The dark side of coproduction: do the costs outweigh the benefits for health research?. Health Res Policy Syst.

[25] Tricco AC, Zarin W, Rios P (2018). Engaging policy-makers, health system managers, and policy analysts in the knowledge synthesis process: a scoping review. Implement Sci.

[26] Moll S, Wyndham-West M, Mulvale G (2020). Are you really doing ‘codesign’? critical reflections when working with vulnerable populations. BMJ Open.

[27] Ovretveit J, Hempel S, Magnabosco JL, Mittman BS, Rubenstein LV, Ganz DA (2014). Guidance for research-practice partnerships (R-PPs) and collaborative research. J Health Organ Manag.

[28] Boaz A, Hanney S, Borst R, O’Shea A, Kok M (2018). How to engage stakeholders in research: design principles to support improvement. Health Res Policy Syst.

[29] Heaton J, Day J, Britten N (2016). Collaborative research and the co-production of knowledge for practice: an illustrative case study. Implement Sci.

[30] Bowen S, Botting I, Graham ID, Huebner LA (2017). Beyond “two cultures”: guidance for establishing effective researcher/health system partnerships. Int J Health Policy Manag.

[31] de Moissac D, Bowen S, Botting I (2019). Evidence of commitment to research partnerships? Results of two web reviews. Health Res Policy Syst.

[32] Bowen S, Botting I, Graham ID (2019). Experience of health leadership in partnering with university-based researchers in Canada: a call to “re-imagine” research. Int J Health Policy Manag.

[33] Greenhalgh T (2020). Bridging the ‘two cultures’ of research and service: can complexity theory help? Comment on “Experience of health leadership in partnering with university-based researchers in Canada: a call to ‘re-imagine’ research. ” Int J Health Policy Manag.

[34] Cooke J (2020). Building research capacity for impact in applied health services research partnerships comment on “Experience of health leadership in partnering with university-based researchers in Canada: a call to “re-imagine” research. ” Int J Health Policy Manag.

[35] Vindrola-Padros C (2020). Can we re-imagine research so it is timely, relevant and responsive? Comment on “Experience of health leadership in partnering with university-based researchers in Canada: a call to ‘re-imagine’ research. ” Int J Health Policy Manag.

[36] Canas E, Shoemaker JK, Kothari A (2020). Promising points for intervention in re-imagining partnered research in health services comment on “Experience of health leadership in partnering with university-based researchers in Canada: a call to ‘re-imagine’ research. ” Int J Health Policy Manag.

[37] Churruca K, Ellis LA, Long JC, Braithwaite J (2020). What can health services researchers offer health systems? Developing meaningful partnerships between academics and health system workers comment on “Experience of health leadership in partnering with university-based researchers in Canada: a call to ‘re-imagine’ research. ” Int J Health Policy Manag.

[38] Plamondon KM (2020). Reimagining researchers in health research comment on “Experience of health leadership in partnering with university-based researchers in Canada: a call to ‘re-imagine’ research. ” Int J Health Policy Manag.

[39] Holmes BJ (2020). Re-imagining research: a bold call, but bold enough? Comment on “Experience of health leadership in partnering with university-based researchers in Canada: a call to ‘re-imagine’ research. ” Int J Health Policy Manag.

[40] Kreindler SA (2020). When coproduction is unproductive comment on “Experience of health leadership in partnering with university-based researchers in Canada: a call to ‘re-imagine’ research. ” Int J Health Policy Manag.

[41] Woodill G, Willi V. Independent Living and Participation in Research: A Critical Analysis. Toronto: Centre for Independent Living in Toronto (CILT); 2006.

[42] Abma TA (2005). Patient participation in health research: research with and for people with spinal cord injuries. Qual Health Res.

[43] Tong A, Sainsbury P, Craig J (2007). Consolidated criteria for reporting qualitative research (COREQ): a 32-item checklist for interviews and focus groups. Int J Qual Health Care.

[44] Poucher ZA, Tamminen KA, Caron JG, Sweet SN (2020). Thinking through and designing qualitative research studies: a focused mapping review of 30 years of qualitative research in sport psychology. Int Rev Sport Exerc Psychol.

[45] Nowell L (2015). Pragmatism and integrated knowledge translation: exploring the compatabilities and tensions. Nurs Open.

[46] Kothari A, McCutcheon C, Graham ID (2017). Defining integrated knowledge translation and moving forward: a response to recent commentaries. Int J Health Policy Manag.

[47] Sparkes AC, Smith B. Qualitative Research Methods in Sport, Exercise and Health: From Process to Product. London: Routledge; 2014.

[48] Adriansen HK (2012). Timeline interviews: a tool for conducting life history research. Qual Stud.

[49] Riessman CK. Narrative Methods for the Human Sciences. London: SAGE Publications; 2008.

[50] Smith B. Narrative analysis in sport and exercise: how can it be done? In: Smith B, Sparkes AC, eds. Handbook of Qualitative Research in Sport and Exercise. London: Routledge; 2016.

[51] Smith B, McGannon KR (2018). Developing rigor in qualitative research: problems and opportunities within sport and exercise psychology. Int Rev Sport Exerc Psychol.

[52] Bass BM, Riggio RE. Transformational Leadership. 2nd ed. New York: Psychology Press; 2005. 10.4324/9781410617095.

[53] Deci EL, Ryan RM. Handbook of Self-Determination Research. Rochester, NY: University of Rochester Press; 2002.

[54] McKevitt C (2013). Experience, knowledge and evidence: a comparison of research relations in health and anthropology. Evid Policy.

[55] Boaz A, Biri D, McKevitt C (2016). Rethinking the relationship between science and society: has there been a shift in attitudes to Patient and Public Involvement and Public Engagement in Science in the United Kingdom?. Health Expect.

[56] Ward F, Popay J, Porroche-Escudero A (2020). Mainstreaming public involvement in a complex research collaboration: a theory-informed evaluation. Health Expect.

[57] O’Mara-Eves A, Brunton G, McDaid D (2013). Community engagement to reduce inequalities in health: a systematic review, meta-analysis and economic analysis. Public Health Res.

[58] Peirson L, Ciliska D, Dobbins M, Mowat D (2012). Building capacity for evidence informed decision making in public health: a case study of organizational change. BMC Public Health.

[59] Morgenroth T, Ryan MK, Peters K (2015). The motivational theory of role modeling: how role models influence role aspirants’ goals. Rev Gen Psychol.

[60] Rogers EM. Diffusion of innovations. New York: Free Press; 2003.

[61] Bandura A. Social Foundations of Thought and Action: A Social Cognitive Theory. Prentice Hall; 1987.

[62] Smith B, Tomasone JR, Latimer-Cheung AE, Martin Ginis KA (2015). Narrative as a knowledge translation tool for facilitating impact: translating physical activity knowledge to disabled people and health professionals. Health Psychol.

[63] Bourbonnais A, Michaud C (2018). Once upon a time: storytelling as a knowledge translation strategy for qualitative researchers. Nurs Inq.

[64] Palmer VJ (2020). The participatory zeitgeist in health care: it is time for a science of participation. J Particip Med.

[65] Nilsen P (2015). Making sense of implementation theories, models and frameworks. Implement Sci.

[66] Esmail R, Hanson HM, Holroyd-Leduc J (2020). A scoping review of full-spectrum knowledge translation theories, models, and frameworks. Implement Sci.

[67] Jull JE, Davidson L, Dungan R, Nguyen T, Woodward KP, Graham ID (2019). A review and synthesis of frameworks for engagement in health research to identify concepts of knowledge user engagement. BMC Med Res Methodol.

[68] Jagosh J, Macaulay AC, Pluye P (2012). Uncovering the benefits of participatory research: implications of a realist review for health research and practice. Milbank Q.

[69] Shippee ND, Domecq Garces JP, Prutsky Lopez GJ (2015). Patient and service user engagement in research: a systematic review and synthesized framework. Health Expect.

[70] Holmes BJ, Best A, Davies H (2017). Mobilising knowledge in complex health systems: a call to action. Evid Policy.

