# Supplementary material for: Researchers’ and Research Users’ Experiences With and Reasons for Working Together in Spinal Cord Injury Research Partnerships: A Qualitative Study
Source: Int J Health Policy Manag. 2021 May 11;11(8):1401–12. doi: 10.34172/ijhpm.2021.35 (PMC9808362; doi:10.34172/ijhpm.2021.35)
Supplement: Supplementary file 1 — Research Team and Study Procedures. [file ijhpm-11-1401-s001.pdf]

Article title: Researchers' and Research Users' Experiences With and Reasons for Working Together in Spinal Cord Injury Research Partnerships: A Qualitative Study

Journal name: International Journal of Health Policy and Management (IJHPM)

Authors' information: Femke Hoekstra[1,2]*, Lee Schaefer[3], Peter Athanasopoulos[4], SCI Guiding Principles Consensus Panel#, Heather L. Gainforth[1,2]

[1]School of Health and Exercise Sciences, University of British Columbia, Kelowna, BC, Canada.

[2]International Collaboration on Repair Discoveries (ICORD), University of British Columbia, Vancouver, BC, Canada.

[3]Department of Kinesiology and Physical Education, McGill University, Montreal, QC, Canada.

[4]Spinal Cord Injury Ontario, Toronto, ON, Canada.

#Members of the SCI Guiding Principles Consensus Panel are listed in the Acknowledgments

(*Corresponding author: heather.gainforth@ubc.ca)

Supplementary file 1: Research Team and Study Procedures

Research team and reflexivity

Dr. Femke Hoekstra is postdoctoral research fellow at the University of British Columbia under supervision of Dr. Heather Gainforth. Dr. Hoekstra completed her PhD on the national implementation of a physical activity promotion program in Dutch rehabilitation. In the context of her PhD project, she has worked together with a diverse group of rehabilitation professionals. She has also clinical experiences with working with people with SCI. Dr. Hoekstra has experiences with and is trained in qualitative research. Dr. Hoekstra's postdoctoral research focused on the development of guiding principles for conducting and disseminating research in partnership with the SCI community. The current interview study is initiated in the context of the IKT guiding principle project[1]. Dr. Hoekstra interviewed all participants.

Dr. Lee Schaefer is Associate Professor in Kinesiology and Physical Educational at McGill University. His research areas include physical education pedagogy, Indigenous health and wellnes. Dr Schaefer is expert in qualitative research methods, with a particular focus on narrative approaches. Dr. Schaefer is also member of the IKT Guiding Principles Panel[1].

Peter Athanasopoulos is Director of Advocacy at SCI Ontario. He has lived experience in SCI. Peter Athanasopoulos has been involved as a research user in many SCI research projects. He is also member of the IKT Guiding Principles Consensus Panel and IKT Guiding Principles Panel[1].

Dr. Heather Gainforth is Associate Professor at the School of Health and Exercise Sciences at the University of British Columbia and a Principal Investigator with the International Collaboration on Repair Discoveries. Dr. Gainforth has expertise in behaviour change, knowledge translation processes, and research partnership approaches. She has strong collaborations with SCI community, illustrated by the many research projects that she has conducted in partnership with SCI research users. Dr. Gainforth is the lead of the IKT Guiding Principles Panel[1].

Data collection

The consent form, survey and interview guide are published on OSF (https://osf.io/n5r4h/). Dr. Hoekstra pilot tested the first draft of the interview guide with Dr. Gainforth. Based on this pilot interview, changes were made to the interview guide in collaboration with Dr. Lee Schaefer and panel members. The final interview guide for researchers included questions as: "Could you tell me a little bit more about how you came to be an academic researcher?", "What moments or experiences have you had that lead you to see research as important?", "What moments or

[1] See: www.IKTprinciples.com

experiences have you had that lead you to being someone interested in SCI research?", and "What moments or experiences have you had that lead you to being someone who sees value in engaging people from the SCI community in the research?". In addition, the interviewee asked probing questions related to moments or experiences listed on the timeline. Examples probing questions included "How did the feel during this moment or experience?", "Have you perceived any barriers or tensions during this moment or experience?", "Have you perceived any successes during this moment or experience?". Furthermore, clarification or curiosity-driven questions were used to further enrich the data.

All interviews were conducted using an online videoconference interface (Vidyo, https://www.vidyo.com). The timeline with participants' moments and experiences was constructed in Powerpoint and shared with the participants via "share screen".

Two participants did not complete the preparation task prior to the interview session. For these two participants, we did not create a timeline prior to the session. Alternatively, participants' CVs were used to guide the interview session.

We did not return transcripts to participants for comments and/or correction. Instead, we did an ethical check with our participants, in which we asked them to review the result section of the manuscript and to provide their permission to include their quotes and related interpretation on their quotes in the manuscript. Participants had the opportunity to make edits to the text, but we did not ask for any alternative interpretation of the data and/or feedback on the content. Some participants were also member of the SCI Guiding Principles Panel. In these cases, participants had the opportunity to review the full manuscript and provide their feedback.
