# Supplementary material for: Researchers’ and Research Users’ Experiences With and Reasons for Working Together in Spinal Cord Injury Research Partnerships: A Qualitative Study
Source: Int J Health Policy Manag. 2021 May 11;11(8):1401–12. doi: 10.34172/ijhpm.2021.35 (PMC9808362; doi:10.34172/ijhpm.2021.35)
Supplement: Supplementary file 3 — Members of the SCI Guiding Principles Panel. [file ijhpm-11-1401-s003.pdf]

Article title: Researchers' and Research Users' Experiences With and Reasons for Working Together in Spinal Cord Injury Research Partnerships: A Qualitative Study

Journal name: International Journal of Health Policy and Management (IJHPM)

Authors' information: Femke Hoekstra[1,2]*, Lee Schaefer[3], Peter Athanasopoulos[4], SCI Guiding Principles Consensus Panel#, Heather L. Gainforth[1,2]

[1]School of Health and Exercise Sciences, University of British Columbia, Kelowna, BC, Canada.

[2]International Collaboration on Repair Discoveries (ICORD), University of British Columbia, Vancouver, BC, Canada.

[3]Department of Kinesiology and Physical Education, McGill University, Montreal, QC, Canada.

[4]Spinal Cord Injury Ontario, Toronto, ON, Canada.

#Members of the SCI Guiding Principles Consensus Panel are listed in the Acknowledgments

(*Corresponding author: heather.gainforth@ubc.ca)

Supplementary file 3: Members of the SCI Guiding Principles Panel

Panel member	Organization(s)	Roles and/or background
Kim Anderson	NASCIC	Researcher + person with lived experience of SCI
Hugh Anton	ICORD, UBC	Researcher + clinician
Peter Athanasopoulos	SCI Ontario, Ontario SCI Solutions Alliance	Leadership role in SCI community organization + person with lived experience of SCI
John Chernesky	Praxis Research Institute, NASCIC	Leadership role in funding organization + person with lived experience of SCI
Susan Forwell	ICORD, UBC	Clinician + researcher
Jocelyn Maffin	SCI BC	Representative from community organisation + person with lived experience of SCI
Kathleen Martin Ginis	ICORD, UBC	Researcher
Christopher B McBride	SCI Canada, SCI BC	Executive director of a community organization
W. Ben Mortenson	ICORD, UBC	Researcher with clinical background
Rhonda Willms	ICORD, GF Strong, UBC	Clinician + researcher

Notes: The SCI Guiding Principles Consensus Panel was established in 2017. NASCIC = North American SCI Consumer Consortium; ICORD = International Collaboration on Repair Discoveries; UBC = University of British Columbia; SCI = Spinal Cord Injury;
