# Supplementary material for: Researchers’ and Research Users’ Experiences With and Reasons for Working Together in Spinal Cord Injury Research Partnerships: A Qualitative Study
Source: Int J Health Policy Manag. 2021 May 11;11(8):1401–12. doi: 10.34172/ijhpm.2021.35 (PMC9808362; doi:10.34172/ijhpm.2021.35)
Supplement: Supplementary file 4 — Research User Engagement in the Qualitative Research Study. [file ijhpm-11-1401-s004.pdf]

Article title: Researchers' and Research Users' Experiences With and Reasons for Working Together in Spinal Cord Injury Research Partnerships: A Qualitative Study

Journal name: International Journal of Health Policy and Management (IJHPM)

Authors' information: Femke Hoekstra[1,2]*, Lee Schaefer[3], Peter Athanasopoulos[4], SCI Guiding Principles Consensus Panel#, Heather L. Gainforth[1,2]

[1]School of Health and Exercise Sciences, University of British Columbia, Kelowna, BC, Canada.

[2]International Collaboration on Repair Discoveries (ICORD), University of British Columbia, Vancouver, BC, Canada.

[3]Department of Kinesiology and Physical Education, McGill University, Montreal, QC, Canada.

[4]Spinal Cord Injury Ontario, Toronto, ON, Canada.

#Members of the SCI Guiding Principles Consensus Panel are listed in the Acknowledgments

(*Corresponding author: heather.gainforth@ubc.ca)

Supplementary file 4: Research User Engagement in the Qualitative Research Study

The research question related to developing guiding principles for Integrated Knowledge Translation (IKT) derived from members of the Spinal Cord Injury (SCI) Guiding Principles Consensus Panel. The table below provides an overview of research activities, associated dates, topics discussed, panel members' concerns and suggestions, and our responses.

Table S3.1: Engagement of the SCI Guiding Principles Consensus Panel in the Review Process

Research activity	Date	Topics discussed	Concerns and suggestions from panel	Our responses
Conceptual design	13 Sept 2017	One-day meeting to establish the consensus panel. During this meeting, the need for a literature review focusing on principles and strategies of research partnership was discussed and outlined as a priority for the panel.	-	-
	May 2018	An online survey was sent to panel members, in which panel members were asked to review participants' eligibility criteria and a first draft of the interview guide.	Panel members provided specific editorial suggestions on the interview guide and added additional questions.	We revised the interview guide and conducted a pilot interview.
Recruitment process	May 2018	An online survey was sent to the panel. Panel members were also asked to provide names of potential participants for the study.	Panel members listed names of potential participants (researchers and research users). Panel members also suggested to recruit via snowballing procedures.	We selected participants from this list.
Before starting data collection	24 August 2018	Conference call to discuss and approve the change in interview guide (introduction of the narrative approach). The lead authors explained that they wanted to use a narrative approach. Panel members did not review the updated/final interview guide, because some panel members were also invited as participants in the study.	All team members agreed with the updates	
Data analysis, interpretation, and dissemination of results.	January 2020	Panel members and co-authors approved final version of the manuscript.	Panel members provided editorial feedback.	

IKT = Integrated Knowledge Translation; SCI = Spinal Cord Injury
