# Supplementary material for: Researchers’ and Research Users’ Experiences With and Reasons for Working Together in Spinal Cord Injury Research Partnerships: A Qualitative Study
Source: Int J Health Policy Manag. 2021 May 11;11(8):1401–12. doi: 10.34172/ijhpm.2021.35 (PMC9808362; doi:10.34172/ijhpm.2021.35)
Supplement: Supplementary file 5 — Role of Critical Friends in the Analyses Process. [file ijhpm-11-1401-s005.pdf]

Article title: Researchers' and Research Users' Experiences With and Reasons for Working Together in Spinal Cord Injury Research Partnerships: A Qualitative Study

Journal name: International Journal of Health Policy and Management (IJHPM)

Authors' information: Femke Hoekstra[1,2]*, Lee Schaefer[3], Peter Athanasopoulos[4], SCI Guiding Principles Consensus Panel#, Heather L. Gainforth[1,2]

[1]School of Health and Exercise Sciences, University of British Columbia, Kelowna, BC, Canada.

[2]International Collaboration on Repair Discoveries (ICORD), University of British Columbia, Vancouver, BC, Canada.

[3]Department of Kinesiology and Physical Education, McGill University, Montreal, QC, Canada.

[4]Spinal Cord Injury Ontario, Toronto, ON, Canada.

#Members of the SCI Guiding Principles Consensus Panel are listed in the Acknowledgments

(*Corresponding author: heather.gainforth@ubc.ca)

Supplementary file 5: Role of Critical Friends in the Analyses Process.

The table below outlines key topics discussed during reflective discussions between first author and co-authors, who acted as critical friends.

Activity	Who	Reflective discussions and key decisions
Discussions of narrative summaries	FH + HG	Lead authors discussed narrative summaries of participants, in which HG asked critical questions about data and provided interpretation. HG provided several suggestions for several theories/framework and relevant literature that could be linked to the data (e.g., transformational leadership theory and social cognitive theory).<br>HG + FH decided to not present narrative types in the manuscript, as that would potentially breach participants' confidentiality.
Discussion of narrative approaches	FH + LS	FH + LS discussed findings and options to organize and present the narrative data.
Discussions of threads and sub-threads	FH + HG	Lead authors discussed identified threads and sub-threads, and decided to link the data to components of the transformational leadership theory.
Discussions of threads and sub-threads	FH + LS	First author discussed the identified threads, sub-threads, and the link with transformational leadership theory with LS. LS mentioned that the link with the theory was not very clear from the presented data.
Discussions of threads and sub-threads	FH + PA	First author discussed the findings with PA (community partner). Figure 1 guided the discussion. PA suggested to distinguish findings between researchers, research users and people with SCI.
Refinement of threads and sub-threads	FH + HG	Lead authors re-organized the threads and sub-threads using pieces of papers (with quotes and threads) and scissors. HG + FH discussed potential capacity building strategies for research partnerships based on the findings and decided to focus the discussion section on these potential capacity building strategies.
Refinement of threads and sub-threads	FH + LS	FH + LS discussed the findings and re-ordered the threads. LS provided specific suggestions to improve the structure of the result section in a way that it better aligned with our narrative approach. LS also provided suggestions to clarify Figure 1.
Finalizing manuscript	All authors	All co-authors provided feedback and editorial changes to the manuscript via email.
